# Natural Killer Cells Are Present in Rag1^−/−^ Mice and Promote Tissue Damage During the Acute Phase of Ischemic Stroke

**DOI:** 10.1007/s12975-021-00923-3

**Published:** 2021-06-08

**Authors:** Leoni Rolfes, Tobias Ruck, Christina David, Stine Mencl, Stefanie Bock, Mariella Schmidt, Jan-Kolja Strecker, Steffen Pfeuffer, Andreas-Schulte Mecklenbeck, Catharina Gross, Michael Gliem, Jens Minnerup, Michael K. Schuhmann, Christoph Kleinschnitz, Sven G. Meuth

**Affiliations:** 1 Department of Neurology With Institute of Translational Neurology, University of Muenster, Albert-Schweitzer-Campus 1, 48149 Muenster, Germany; 2 Department of Neurology, University Hospital Düsseldorf, Heinrich-Heine-University Düsseldorf, Düsseldorf, Germany; 3 Department of Neurology, University Hospital Essen, University Duisburg-Essen, Essen, Germany; 4 Center for Translational Neuro- and Behavioral Sciences (C-TNBS), University Hospital Essen, University Duisburg-Essen, Essen, Germany; 5 Department of Neurology, University Hospital Würzburg, Würzburg, Germany

**Keywords:** Infarction, Middle cerebral artery occlusion, Animal model, Inflammation, Natural killer cells

## Abstract

Rag1^−/−^ mice, lacking functional B and T cells, have been extensively used as an adoptive transfer model to evaluate neuroinflammation in stroke research. However, it remains unknown whether natural killer (NK) cell development and functions are altered in Rag1^−/−^ mice as well. This connection has been rarely discussed in previous studies but might have important implications for data interpretation. In contrast, the NOD-Rag1^null^IL2rg^null^ (NRG) mouse model is devoid of NK cells and might therefore eliminate this potential shortcoming. Here, we compare immune-cell frequencies as well as phenotype and effector functions of NK cells in Rag1^−/−^ and wildtype (WT) mice using flow cytometry and functional in vitro assays. Further, we investigate the effect of Rag1^−/−^ NK cells in the transient middle cerebral artery occlusion (tMCAO) model using antibody-mediated depletion of NK cells and adoptive transfer to NRG mice in vivo. NK cells in Rag1^−/−^ were comparable in number and function to those in WT mice. Rag1^−/−^ mice treated with an anti-NK1.1 antibody developed significantly smaller infarctions and improved behavioral scores. Correspondingly, NRG mice supplemented with NK cells were more susceptible to tMCAO, developing infarctions and neurological deficits similar to Rag1^−/−^ controls. Our results indicate that NK cells from Rag1^−/−^ mice are fully functional and should therefore be considered in the interpretation of immune-cell transfer models in experimental stroke. Fortunately, we identified the NRG mice, as a potentially better-suited transfer model to characterize individual cell subset-mediated neuroinflammation in stroke.

**Supplementary Information:**

The online version contains supplementary material available at 10.1007/s12975-021-00923-3.

## Introduction

Stroke continues to be one of the leading causes of death and disability [1]. Increasing evidence indicates that early and time-delayed inflammatory processes are critical variables that determine the extent of neuronal disintegration and regeneration [2]. Transfer animal models have been widely established in experimental stroke research to characterize the effect of individual immune-cell subsets and specific signaling pathways [3]. Rag1^−/−^ mice, devoid of mature T and B cells, represent one of the most extensively used mouse models to investigate immunological questions related to stroke development [3–5].

Of note, natural killer (NK) cells have been described to play multiple roles in ischemic stroke, being associated with post-stroke inflammation [6, 7], immunodepression, and infections in both animal models [7] and humans studies [8, 9]. NK cells are abundant in ischemic brain tissue in stroke animal models [6, 7] and post-mortem brain tissues from stroke patients [7]. They are swiftly mobilized during the earliest phases of immune responses, with kinetic experiments in rodents showing that NK cells accumulate in the brains as early as 3 h after transient middle cerebral artery occlusion (tMCAO) and peak at days 1 [10] to 3 [7] after stroke onset. NK cells exert cytotoxic or cytolytic effects on target cells, but also interact with other cell types (including CNS resident cells [7, 11, 12] and other immune cells [13]) to influence the stroke progress. They represent the closest innate immune cell lineage to adaptive immune cell populations [14]. Under certain circumstances, T cells even acquire NK cell-like properties characterized by the expression of NK cell immunoreceptors. Contrastingly, potential adaptive immune features displayed by other innate immune cell types have so far not been described [15]. These last discoveries should prompt a re-evaluation whether persisting NK cells in adoptive transfer mouse models might interfere with immune-cell subset-specific analysis, especially of T und B cells. In regard to the Rag1^−/−^ adoptive mouse model, the arrest of B and T cell differentiation occurs at an early stage and correlates with the inability to perform rearrangement of the V, D, and J gene segments of the antigen receptor, and generate receptor diversity (V(D)J recombination) [16, 17]. However, innate lymphocyte lineages such as NK cells are thought to express germline-encoded antigen receptors and do not require RAG for development and function [18]. Therefore, it is likely that NK cell development and functions are not altered in Rag1^−/−^ mice and probably bias outcomes in stroke models (Supplemental Fig. S1). NOD-Rag1^null^IL2rg^null^ (NRG) mice represent a potential alternative to overcome this shortcoming as they have a combined Rag1 and interleukin (IL)-2 receptor-γ null mutation lacking mature T and B cells as well as NK cells [19, 20].

We here performed a characterization of NK cells derived from Rag1^−/−^ and wildtype (WT) mice, ranging from detailed immunological phenotyping and functional characterizations to in-depth studies in experimental stroke, using the tMCAO mouse model. By using a suitable immunodeficient animal stroke model, we probably gain a more detailed understanding of the underlying mechanisms that shape ischemic immune reactions. This might open new perspectives for translation of novel experimental therapies into an effective treatment for stroke patients and thus contribute to overcome the translational roadblock [2, 21].

## Methods

### Animals

Male and female 8- to 12-week-old C57BL/6 J WT mice were purchased from Charles River Laboratories (Sulzfeld, Germany), age and sex-matched Rag1^−/−^ and NRG mice from The Jackson Laboratories (Bar Harbor, USA). Mice were kept in individually ventilated cage animal housing. We used both male and female mice in terms of immunological characterization (e.g., functional assays, immunophenotyping assays, and as donors for adoptive transfer experiments). Experimental stroke was only performed with male mice. Two hundred and ninety-seven animals were used in this study, including all tMCAO surgeries, functional assays, immunophenotyping assays, as well as adoptive transfer experiments. A total of 245 animals were included in the final analysis. Four animals suffered complications during surgery, 6 animals died before tissue preparation, 3 animals were excluded because of cerebral hemorrhage, 7 animals used for flow cytometric analysis had to be excluded due to technical problems in tissue preparation, and 32 animals were used as donor for the adoptive transfer experiments. Experiments were performed in accordance with animal welfare regulations, and experimental protocols were approved by the local governmental authorities (Regierung von Unterfranken, Bavaria, Germany (55.2–2531.01–73/13) and Landesamt für Natur, Umwelt und Verbraucherschutz, North Rhine-Westphalia, Germany (81–02.04.2018.A127)).

### Ischemia Model—Transient Middle Cerebral Artery Occlusion (tMCAO)

Focal cerebral ischemia was induced by 60-min tMCAO, as described previously [22]. Briefly, tMCAO was induced under inhalation anesthesia (isoflurane, 2%) using the intraluminal filament (6021PK10; Doccol Company) technique, in a blinded fashion. Thereby, a monofilament was inserted via the common carotid artery into the middle cerebral artery (MCA) which leads to reproducible infarcts. After 60 min, the filament was withdrawn to allow reperfusion.

All stroke experiments were performed following the ARRIVE guidelines (https://www.nc3rs.org.uk/arrive-guidelines). Mice were randomly assigned to the operators by an independent person not involved in data analysis. Surgery and evaluation of all readout parameters were performed in a blinded manner.

### Tissue Preparation and Infarct Volume Assessment

One to 7 days after tMCAO, mice were perfused through the left ventricle with phosphate-buffered saline under isoflurane anesthesia. In detail, incisions were performed along the thoracic midline from just below the xiphoid process to the clavicle and the xiphoid process along the base of the ventral thorax laterally to expose the thoracic field completely. After cutting through the thoracic musculature and ribcage between the breastbone and medial rib insertion points, we separated the diaphragm from the chest wall on both sides with scissor cuts. We gently grasped the pericardium and cut the right atrium with scissors. Next, we punctured the left ventricle and continued perfusion until the fluid exiting the right atrium was completely clear (minimum 20 ml phosphate-buffered saline).

Directly after cardiac perfusion, brains were removed and cut into four 2-mm-thick coronal sections. Slices were stained for 20 min at 37 °C with 2% 2,3,5-triphenyltetrazolium chloride to visualize infarctions [23]. Edema-corrected infarct volumes were calculated by planimetry (ImageJ software; National Institutes of Health).

### Functional Outcome Tests

After stroke induction, we repeatedly scored every mouse immediately after reawakening and every day until sacrifice, using the following scales: Global neurologic deficits were assessed by applying the Bederson score [24] (scale from 0 to 5: 0 no deficit, 1 preferential turning, 2 circling, 3 longitudinal rolling, 4 no movement, 5 death). The grip test score was used to monitor motor function and coordination. Thereby, the mouse is hanging from a wooden pole, placed between two posts 50 to 60 cm above the ground (scale 0–5: 0 the mouse falls down; 1 the mouse hangs on the wooden pole with one or both front paws; 2 the mouse hangs on the wooden pole with one or both front paws and tries to climb on the wooden pole; 3 the mouse hangs on the wooden pole with one or both front paws and one or both back paws; 4 the mouse hangs on the wooden pole with both front and back paws and wraps its tail around the wooden pole; 5 the mouse hangs on wooden pole with both front and back paws and wraps tail around it and gets to outside rack) [25].

### Cell Isolation

Single-cell suspensions from naïve WT, Rag1^−/−^, and NRG mouse spleens and lymph nodes (LN; cervical) were prepared. Tissues were homogenized and strained through a 40-μm nylon filter (BD Biosciences, Germany). Homogenates were rinsed with washing medium (Dulbecco’s Modified Eagle’s Medium, DMEM, Invitrogen, USA) containing 1% FBS (ScienCell, USA), 1% glutamine (Gibco Life Technologies, USA), and 1% antibiotics (Sigma-Aldrich, USA) and shortly resuspended in erythrocyte lysis buffer (150 mM NH4Cl, 10 mM KHCO3, 0.1 mM EDTA; pH 7.3).

Brain tissues were cut into pieces, homogenized in phosphate-buffered saline, layered on a density gradient using Lymphoprep™ (Fresenius, Germany), and separated by centrifugation for 16 min at 500* g*. After isolating cells, they were washed and resuspended in the respective staining buffer. To quantify numbers of cells isolated from the central nervous system, beads (Beckman Coulter, USA) were added.

### Flow Cytometry

Single cell suspensions were stained for 30 min at 4 °C with the appropriate combination of indicated fluorescence-labeled monoclonal antibodies in phosphate-buffered saline, containing 0.1% sodium azide and 0.1% bovine serum albumin (Sigma-Aldrich, for details see Table 1). Corresponding isotype controls were used for all staining. Flow cytometric analysis of stained cells was performed following standard protocols. Cells were analyzed on a BD FACSCalibur Flow Cytometer (BD Biosciences) or a Gallios Flow Cytometer (Beckman Coulter) using the *Kaluza Analysis Software* (Beckman Coulter) and visualized with *GraphPad Prism* (USA).

**Table 1:** Antibodies used for flow cytometry, NK cell depletion, and immunofluorescence staining

Antigen	Reactivity	Supplier		Order No
Flow cytometry				
CD3	Mouse	BioLegend		100,218
CD4	Mouse	BioLegend		100,531
CD8a	Mouse	BioLegend		100,730
CD11b	Mouse/Human	BioLegend		101,263
CD11c	Mouse	BioLegend		117,306
CD43	Mouse	BioLegend		143,205
CD45	Mouse	BioLegend		103,116
CD27	Mouse	BioLegend		124,215
CD45R(B220)	Mouse	BioLegend		103,208
CD49b	Mouse	BioLegend		108,915
CD62L	Mouse	BioLegend		104,418
CD122	Mouse	Beckman Coulter		732,491
CD107a	Mouse	BioLegend		121,616
DNAM-1	Mouse	BD pharmingen		565,549
KLRG1	Mouse/human	BioLegend		138,415
Ly49A	Mouse	BioLegend		138,703
Ly49D	Mouse	BioLegend		103,708
Ly49H	Mouse	Miltenyi		130–5321-80
NK1.1	Mouse	BioLegend		108,710
NKG2D	Mouse	eBioscience		12–5882-81
Nkp46	Mouse	Miltenyi		130–102-300
NK cell depletion				
NK1.1 (clone PK136)	Mouse, human	Bio X Cell	BE0036	
Immunofluorescence staining				
NKp46	Mouse, human	Abcam	ab233558	
NeuN	Mouse	Millipore	MAB377	

### Natural Killer Cell Depletion by Anti-NK1.1-antibody in vivo (PK136)

NK cells are defined as CD3^−^NK1.1^+^ cells in mice. NK.1.1 (killer cell lectin-like receptor subfamily B, CD161) is referred to as “pan-NK cell marker” [26]. For in vivo depletion of NK1.1 expressing cells, mice were injected intraperitoneally (i.p) every second day with 300 µg of NK1.1-specific monoclonal antibody (clone PK136 (Bio X Cell, USA)). The control group was given similar doses of the respective isotype (IgG2, clone 2A3; BioXCell). The dosage was chosen in accordance with previous published studies that used the same antibody [27, 28]. We confirmed the depletion by the presence of ≤ 0.5% NK1.1^+^ cells among all leukocytes in the spleen and lymph nodes 24 h to 7 days after ischemia, by flow cytometric analysis.

### CD107a (LAMP-1) Degranulation Assay

CD107a expression on NK cells was measured to analyze NK cell degranulation [29]. To measure the degranulation response by staining with anti-CD107a during target cell stimulation, NK cells were incubated with either YAC or P815 target cells (co-incubated with NKG2D) at a 1 to 1 ratio of 3 × 10^5^ cells/well in medium containing anti-mouse CD107a or isotype control (Biolegend). The assay was incubated for 5 h at 37 °C/5%CO_2_. Cells were washed and stained with anti-NK1.1 and anti-CD3 antibodies. Using flow cytometry, the CD107a positive rate of CD3^−^NK1.1^+^ NK cells was analyzed.

### Adoptive Transfer Experiments

NK cells were isolated from spleen and LN cell suspensions of either WT or Rag1^−/−^ mice. For the enrichment of NK cells derived from WT mice, we performed magnetic bead-based separation (anti-NK.1.1 isolation kit, mouse, Miltenyi, Germany) prior to flow cytometric cell sorting (staining for CD3 and NK.1.1). The MACS cell separation was performed according to the supplier’s manual. Cells were resuspended to 1,000,000 cells per 100μL in phosphate-buffered saline and subsequently injected intravenously (by tail vein injections) in NRG mice. NK cell-reconstituted NRG mice were subjected to tMCAO 24 h after injection. A purity of ≥ 90% was achieved in all experiments. The control group received 100 µl phosphate-buffered saline without NK cells.

### Immunofluorescence Staining of NK Cells

Twenty-four hours after tMCAO, WT and Rag1^−/−^ mice were perfused through the left ventricle with phosphate-buffered saline for 5 min and 4% paraformaldehyde for 10 min under deep isoflurane anesthesia. Brains were removed, fixed with 4% paraformaldehyde overnight, and immersed in 30% sucrose for 3 days. Mounted coronal cryosections were rinsed in 3% H_2_O_2_/Methanol for 10 min to block endogenous peroxidases and thereafter incubated with blocking reagent (Roche Diagnostics) for 15 min to prevent unspecific protein binding. Subsequently, we used the following primary antibodies: anti-Nkp46 (NCR1, 1:200, Abcam, ab233558, Cambridge, UK) and anti-NeuN (1:100, Millipore). NeuN was directly visualized with anti-mouse-488 (1:150, Life). To amplify the signal of Nkp46, we applied HRP-conjugated streptavidin (1:200, DAKO Denmark) and biotinyl tyramide (1:200, 15 min, RT) after incubation with the biotinylated secondary antibody goat-anti-rabbit (1:200). Afterwards, the amplified antigen was visualized with streptavidin-conjugated dye (Alexa Fluor594, 1:200, Molecular Probes). For nuclear counterstaining, we applied a mounting medium with DAPI (Vector, Burlingame). Images were taken with a fluorescence microscope (Nikon Eclipse 80i, Nikon), and confocal fluorescence z-stacks were taken with Zeiss AxioVision ImagerM2, Zeiss. Nkp46-positive NK cells of the whole ischemic hemisphere were counted in two coronal cryosections of each animal, and the mean per animals was calculated.

### Statistical Analysis

Prior to conduction of analyses, we determined the necessary sample size to detect an alteration of at least 33% regarding splenocyte composition in Rag1^−/−^ mice compared to WT animals. A two-tailed test at an alpha level of 0.05 and a 1-beta of 0.80 based on the abovementioned assumption resulted in an effect size of d = 1.65. Hence, a minimum sample size of 6 animals per group was determined for immunophenotyping studies. Further analyses derived from these animals however were not corrected for multiple testing given the explorative setup of these tests. Regarding tMCAO experiments, we calculated a group size of 10 animals per group to detect significant differences in infarct volume between mice subjected to NK cell depletion/substitution and controls, using an alpha level of 0.05, a 1-beta of 0.80, and an effect size of d = 1.25.

Results are displayed as mean ± SEM unless indicated otherwise. Statistical analysis comparing two groups was performed using then unpaired Student’s t-test for or the Mann–Whitney rank sum test where appropriate. The one-way ANOVA including Bonferroni’s post hoc and the Kruskal–Wallis test including Dunn’s post hoc test were used for comparison of more than two groups where appropriate. The level of significance was labeled according to the p-values (* p < 0.05, ** p < 0.01, or *** p < 0.001).

## Results

### Characterization of NK cells from Rag1^−/−^ mice

In the first set of experiments, we examined the immunological phenotype of Rag1^−/−^ mice in particular referring to NK cells. Therefore, we assessed the absolute and relative cell numbers of LN and spleen tissue (as both represent secondary lymphoid organs) with flow cytometry.

**Fig. 1:**
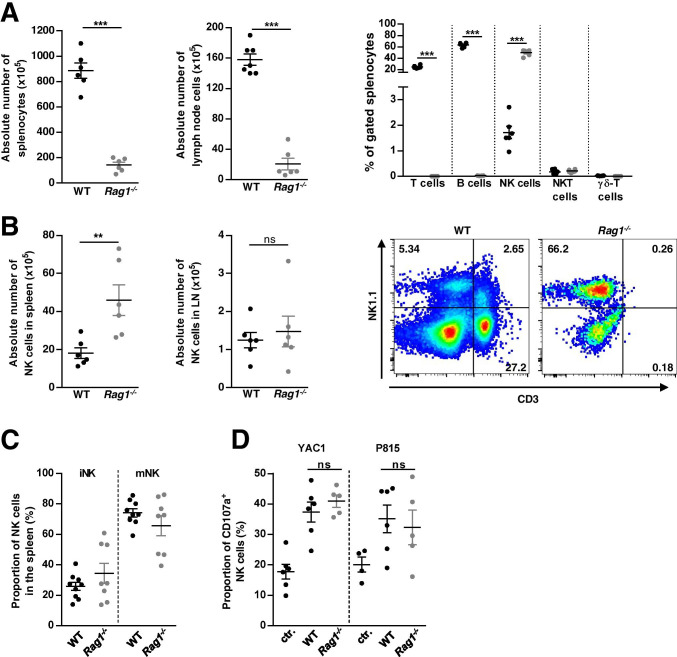
Immunological characterization of naïve Rag1^−/−^ mice. **A** Absolute number of peripheral immune cells, in spleen and lymph node (LN), as well as proportion of gated splenocytes of wildtype (WT, C57BL/6) and Rag1^−/−^ mice assessed by single-cell suspension cell counting. **B** Absolute number of natural killer (NK) cells in spleen and LN of WT and Rag1^−/−^ mice. Exemplary flow cytometric staining of LN cells derived from WT and Rag1^−/−^ mice gated for T cells (CD3^+^NK1.1^−^), NKT cells (CD3^+^NK1.1^+^), and NK cells (CD3^−^NK1.1^+^) is outlined. **C** Proportion of NK cell subsets (immature NK (iNK) and mature NK (mNK) cells) in spleen of WT and Rag1^−/−^ mice, with no differences between the respective mouse strains. **D** Lysis of YAC1 and P815 target cells by NK cells derived from WT and Rag1^−/−^ mice, assessed as the proportion of CD107a^+^ cells; ns, not significant

According to previous studies, we found significantly reduced absolute cell numbers in spleen and LN of Rag1^−/−^ compared to WT mice (Fig. 1A, n = 6, *p* < *0.001*) [3, 16]*.* Detailed analysis of lymphocyte subsets revealed that Rag1^−/−^ mice completely lack mature T cells and B cells (Fig. 1A, n = 6, *p* < *0.001*); however, they did not show alterations in the relative and absolute number of NK cells (gated as CD3^−^NK1.1^+^) compared to WT mice (Fig. 1A + B, n = 6). On the contrary, Rag1^−/−^ mice even showed an increased absolute number of NK cells in the spleen compared with WT mice (Fig. 1B, n = 6, *p* < *0.01*). Moreover, characterization of NK cell surface marker expression did not show any significant differences comparing NK cells derived from spleen of WT and Rag1^−/−^ mice (Supplement Fig. S2A, WT: n = 9, Rag1^−/−^: n = 8). In the next step, we evaluated NK cell subset distribution using spleen tissue of WT and Rag1^−/−^ mice. We detected no difference regarding the percentage of immature NK (iNK) and mature NK (mNK) cells (Fig. 1C, WT: n = 9 WT, Rag1^−/−^: n = 8) or their surface marker expression (Supplement Fig. S2B + C, WT: n = 9, Rag1^−/−^: n = 8).

To further describe the functionality of Rag1^−/−^ NK cells, we analyzed the release of cytotoxic granules measured by the surface exposure of CD107a, a marker of NK cell activation and cytotoxic degranulation. To this end, the proportion of CD107a^+^ Rag1^−/−^ and WT NK cells was analyzed after stimulation with YAC1 and P815 target cells (co-incubated with NKG2D). NK cells of either group displayed no relevant differences in cytolytic function in vitro (Fig. 1D, WT: n = 6, Rag1^−/−^: n = 5).

In summary, peripheral NK cells of Rag1^−/−^ mice were comparable in number, phenotype, and function to those found in the periphery of WT mice.

### In vivo Relevance of Antibody-Mediated Depletion of WT NK Cells Using an Anti-NK1.1 Antibody

**Fig. 2:**
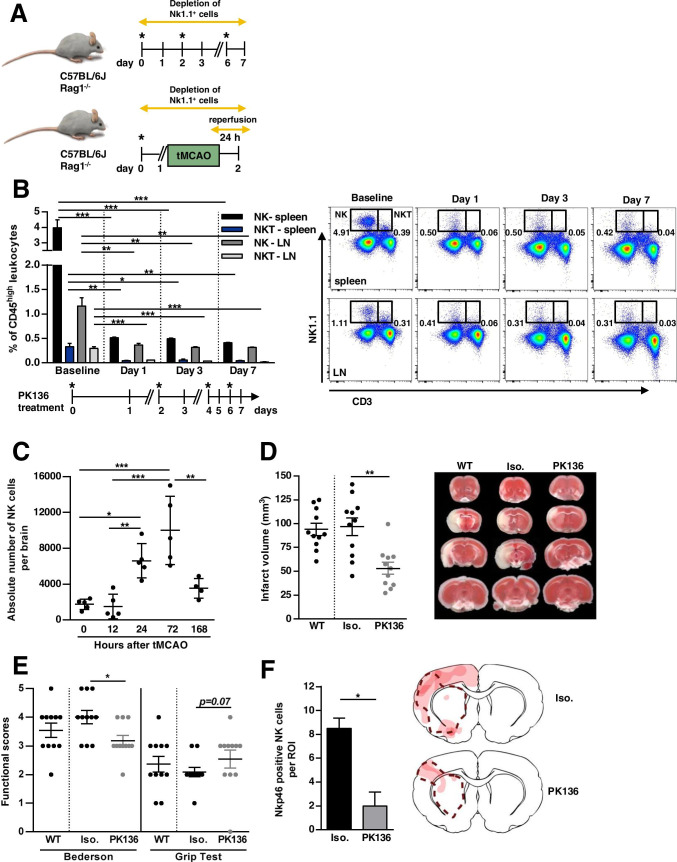
In vivo depletion of natural killer (NK) and natural killer T (NKT) cells with an anti-NK1.1 antibody (PK136). **A** Schematic illustration of in vivo depletion of Nk1.1^+^ cells in wildtype (WT, C57BL/6) and Rag1^−/−^ mice. Upper panel: Mice were injected intraperitoneally every second day with 300 µg of NK1.1-specific monoclonal antibody (clone PK136, *). The control group was given similar doses of the respective isotype. We confirmed the depletion by flow cytometric analysis of leukocyte subpopulations isolated from lymph nodes (LN) and spleen, 24 h to 7 days after first PK136 administration (see Fig. 3B + C). Lower panel: 24 h prior to focal ischemia by transient middle cerebral artery occlusion (tMCAO), mice were injected intraperitoneally with 300 µg of NK1.1-specific monoclonal antibody (*). Ischemia was induced by 60 min of tMCAO. Twenty-four hours after stroke onset, behavioral tests and infarct volume assessment was performed. **B** Sustained effect of NK and NKT cell depletion in spleen and LN of WT mice after administration of PK136 (given as the percentage of CD45^high^ leukocytes). Days of treatment are outlined in the timeline below the figure. Mice were injected intraperitoneally with 300 µg of NK1.1-specific monoclonal antibody (*). Exemplary flow cytometric staining of LN and spleen derived from WT mice gated for T cells (CD3^+^NK1.1^−^), NKT cells (CD3^+^NK1.1^+^), and NK cells (CD3^−^NK1.1^+^) at baseline, and 1, 3, and 7 days after NK1.1^+^ cell depletion is depicted. **C** Time course of NK cell infiltration after 60 min of tMCAO in mice: Flow cytometry of the whole brain derived from WT mice 0, 12, 24, 72, and 168 h post-stroke. **D** Quantification of the infarct volume in WT mice without any treatment (WT) and WT mice treated with either anti-NK1.1 antibody (PK136) or isotype control (Iso.). Representative images of the infarct volume are depicted using 2,3,5-triphenyltetrazolium chloride (TTC) staining. **E** Functionally behavioral scores, namely Bederson and grip test in WT, Iso. and PK136 mice 24 h after focal cerebral ischemia are shown. **F** The number of NK cells in the ischemic hemisphere (region of interest: ROI) of Iso. and PK136 animals 24 h after 60-min tMCAO are compared by immunofluorescence staining of Nkp46 (right). Nkp46-positive NK cells of the whole ischemic hemisphere were counted in two coronal cryosections of each animal, and the mean value of both sections was calculated per animal. Spatial localization of NK cells in the ischemic hemisphere is shown by a heat map (left). The more intense the red coloration, the more NK cells could be found in this area. The dashed line indicates the infarct area in the both treatment groups

To analyze the effect of NK cells in experimental stroke and to evaluate whether these NK cells might bias results of adoptive transfer studies, NK cells were depleted in vivo by using an anti-NK1.1 monoclonal antibody (PK136) [27, 28]. To ensure a depletion of NK1.1^+^ cells for a period of 7 days, dosage and application intervals were tested in a pilot experiment. In line with previous studies [27, 28], repeated injection of 300 µg PK136 every second day (Fig. 2A) significantly depleted NK (CD3^−^NK1.1^+^) and natural killer T cells (NKT) cell (CD3^+^NK1.1^+^) populations in the spleen (baseline NK 3.98 ± 0.50% vs. day 7 NK 0.41 ± 0.003%; *p* < *0.001*; baseline NKT 0.33 ± 0.07% vs. day 7 NKT 0.04 ± 0.007%; *p* < *0.01*) and LN (baseline NK 1.16 ± 0.17% vs. day 7 NK 0.32 ± 0.003%; *p* < *0.01*; baseline NKT 0.29 ± 0.04% vs. day 7 NKT 0.02 ± 0.005%; *p* < *0.001*) of WT mice over a period of 7 days (Fig. 2B, n = 3). Related to the subsequent experiments in acute ischemic stroke, the analysis time points of 24 and 72 h were based on the kinetics of NK cell infiltration in experimental stroke in rodents (with peak at days 1 [10] to 3 [7] after stroke onset). The 7-day value was designed as a long-term value to validate sufficient depletion even over this time period.

To address the relevance of NK cells on stroke-mediated tissue damage, we first examined the spatial and temporal course of NK cell infiltration in the brain of WT mice by flow cytometry, 6, 12, 24, 72, and 168 h after tMCAO induction. The cell number of NK cells significantly increased after stroke, reaching a maximum on day 3 and nearly decreased to baseline at our latest assessment time point at day 7 (Fig. 2C, n = 5, except for the subgroup after 168 h with n = 4, *p* < *0.001*).

**Fig. 3:**
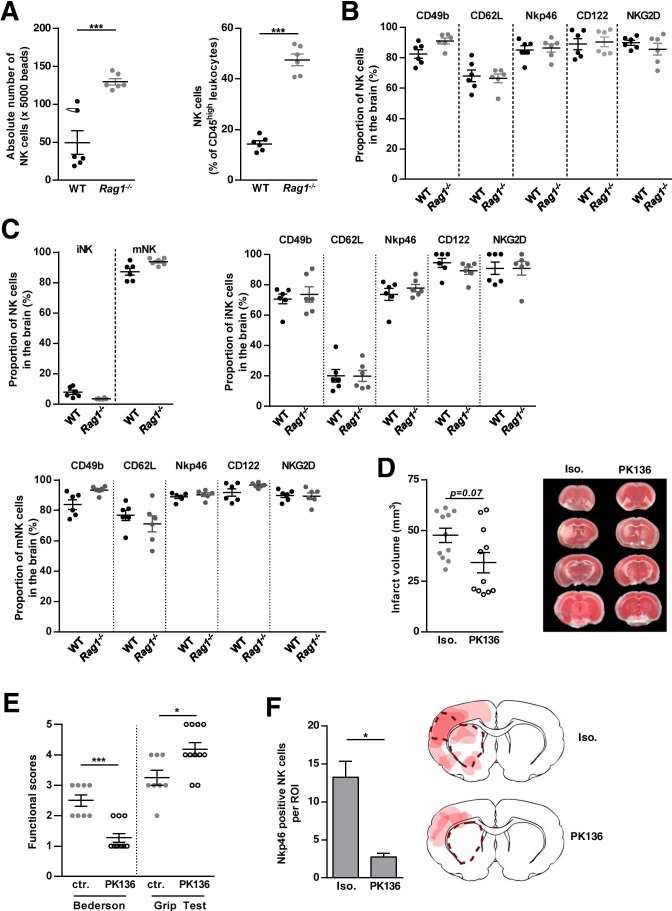
In vivo relevance of Rag1^−/−^ NK cells for stroke development. **A** NK cells in the brain of wildtype (WT, C57BL/6) and Rag1^−/−^ mice determined by flow cytometry: bars represent either the absolute numbers of NK cells per 5.000 beads or the proportion of NK cells to the total number of CD45^high^ leukocytes in WT and Rag1^−/−^ mice. **B** Flow cytometric evaluation of freshly isolated brain tissue from WT and Rag1^−/−^ mice for the indicated NK cell surface markers. **C** Proportion of NK cell subsets (immature NK (iNK) and mature NK (mNK) cells), as well as flow cytometric evaluation of the indicated NK cell surface markers in the brain of WT and Rag1^−/−^ mice. **D** Quantification of the infarct volume in Rag1^−/−^ mice treated with either anti-NK1.1 antibody (PK136) or isotype control (Iso.). Representative images of the infarct volume are depicted using 2,3,5-triphenyltetrazolium chloride (TTC) staining. **E** Functionally behavioral scores, namely, Bederson and grip test, in Iso. and PK136-treated Rag1^−/−^ mice 24 h after focal cerebral ischemia are shown. **F** The number of NK cells in the ischemic hemisphere (region of interest: ROI) of Iso. and PK136-treated Rag1^−/−^ mice 24 h after 60-min tMCAO are compared by immunofluorescence staining of Nkp46 (right). Nkp46-positive NK cells of the whole ischemic hemisphere were counted in two coronal cryosections of each animal, and the mean value of both sections was calculated per animal. Spatial localization of NK cells in the ischemic hemisphere is shown by a heat map (left). The more intense the red coloration, the more NK cells could be found in this area. The dashed line indicates the infarct area in the both treatment groups

Moreover, we determined infarct volumes and functional outcomes 24 h after focal cerebral ischemia in WT mice treated with either PK136 or isotype control. PK136 or isotype control was administered 24 h before stroke induction (Fig. 2A). Notably, NK cell depletion by PK136 significantly reduced infarct volumes in WT (96.73 ± 9.34 mm^3^ vs. 53.05 ± 6.24 mm^3^, *p* < *0.01*, Fig. 2D, n = 11). Of note, the treatment with the isotype did not lead to a change in the infarct volume compared with WT mice without any treatment (96.73 ± 9.34 mm^3^ vs. 93.76 ± 6.35 mm^3^, Fig. 2D, n = 11). The reduction in infarct size after PK136 treatment was functionally relevant, since the Bederson score assessing global neurologic function (3.92 ± 0.23 vs. 3.0 ± 0.18, *p* < *0.05*) and the grip test were significantly better in PK136-treated mice compared to isotype control (2.09 ± 0.16 vs. 2.55 ± 0.31, *p* = *0.067*, Fig. 2D, n = 11). Next, we analyzed the number of NK cells by immunofluorescence staining of Nkp46-positive cells in the ischemic hemisphere (Fig. 2F). Twenty-four hours after tMCAO PK136-treated animals showed significantly reduced NK cells in the region of interest assessed by fluorescence microscopy, compared with isotype controls (8.50 ± 0.86 cells vs. 2.0 ± 1.15 cells, *p* < *0.05*, Fig. 2F, n = 4). Of note, NK cells are found to infiltrate throughout the infarct hemisphere. They are principally localized with the infarcted tissue, but also found in periinfarct areas or ischemic penumbra, in both the cortex and the basal ganglia (for details see heat maps in Figs. 2F and 3F). Moreover, we consistently found NKp46-positive NK cells in close proximity to ischemic neurons or their axons. Occasionally, a NeuN signal was further observed in the Nkp46-positive NK cells (representative immunofluorescence staining of NKp46-positive NK cells is shown in Supplement Figure S3).

In summary, we demonstrated that NK cells of WT mice are likely to be critically involved in early tissue injury during ischemic stroke.

### Phenotype, Function and in vivo Relevance of Rag1^−/−^ NK Cells for Stroke Development

To evaluate whether the observed detrimental effect of NK cells on the WT stroke outcome also applies to the Rag1^−/−^ mouse model, where only NK cells are present, and must therefore be considered when interpreting the results of adoptive transfer stroke models, we further characterized naïve Rag1^−/−^ brain NK cells and their function in stroke development.

We first compared the number and proportion of NK cells in whole brains of naïve Rag1^−/−^ and WT mice. Of note, flow cytometric evaluation showed significantly increased NK cell numbers in the brains of Rag1^−/−^ compared to WT (Fig. 3A, n = 6, *p* < *0.001*). According to the peripheral NK cell surface marker expression (Supplement Fig. S2), the characterization of brain NK cells (including markers of NK cell development (CD49b) and differentiation (CD62L), cell activation (Nkp46, NKG2D), and immune tolerance (CD122)) further did not show any significant differences comparing WT and Rag1^−/−^ mice (Fig. 3B, n = 6). In the next step, we evaluated the NK cell subset distribution using brains of WT and Rag1^−/−^ mice. We detected no difference regarding the percentage of iNK and mNK cells and their surface marker expression, respectively (Fig. 3C, n = 6).

To address the pathophysiologic relevance of Rag1^−/−^ NK cells in early stroke development, Rag1^−/−^ mice were subjected to tMCAO and either treated with PK136 or with isotype control (Fig. 2A). Noteworthy, NK cell depletion by PK136 significantly ameliorated functional stroke outcome (Bederson score 2.50 ± 0.19 vs. 1.27 ± 0.14, *p* < *0.001*; grip test 3.25 ± 0.25 vs. 4.18 ± 0.23, *p* < *0.05*, Fig. 3E, n = 11) in Rag1^−/−^ mice, without reaching significance level in the stroke volume (45.73 ± 4.23 mm^3^ vs. 31.78 ± 4.33 mm^3^, *p* = *0.07*, Fig. 3D, n = 11).

In accordance to the findings in WT mice, PK136-treated Rag1^−/−^ mice showed significantly reduced NK cells in the ischemic hemisphere assessed by fluorescence microscopy, compared with Rag1^−/−^ isotype controls (13.25 ± 2.09 cells vs. 2.75 ± 0.48 cells, *p* < *0.05*, Fig. 3F, n = 4). In terms of spatial distribution, these cells also behaved similar to WT NK cells (Figs. 2F + 3F + Supplement Figure S3).

The effect of PK136 treatment in both WT and Rag1^−/−^ mice indicates a pivotal role of NK cells in stroke formation, which might bias cell subtype-specific analysis in Rag1^−/−^ adoptive transfer experiments. Hence, the question arises, whether there is an NK-cell-free mouse model, potentially better suited.

### Stroke Development in NRG Mice after Adoptive Transfer with WT and Rag1^−/−^ NK Cells

**Fig. 4:**
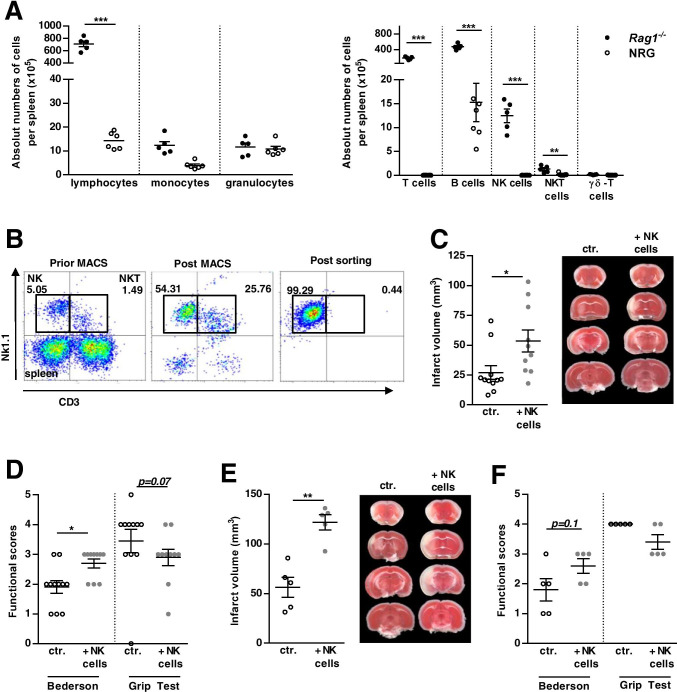
Stroke development in adoptive transfer tMCAO in NRG mice substituted with either WT or Rag1^−/−^ natural killer (NK) cells. **A** Immunological phenotyping of splenocytes derived from naïve NRG mice by flow cytometry. Absolute numbers and proportions of the indicated cell subsets from wildtype (WT, C57BL/6) and NRG mice are outlined. **B** Exemplary flow cytometry of splenocytes derived from WT mice gated for T cells (CD3^+^NK1.1^−^), NKT cells (CD3^+^NK1.1^+^), and NK cells (CD3^−^NK1.1^+^), outlining the purity of NK cell isolation via magnetic (MACS) and flow cytometric cell sorting. **C** WT NK cell adoptive transfer in NRG mice subjected to 60-min tMCAO. Quantification of the infarct volume in NRG mice supplemented with NK cells from either WT mice or control (ctr.). Representative images of the infarct volume are depicted using 2,3,5-triphenyltetrazolium chloride (TTC) staining. **D** Functional behavioral scores, namely, Bederson and grip test, in NK cell-supplemented NRG mice, compared with controls, are shown. **E** Rag1^−/−^ NK cell adoptive transfer in NRG mice subjected to tMCAO. Quantification of the infarct volume is outlined. Representative images of the infarct volume are depicted using 2,3,5-Triphenyltetrazolium chloride (TTC) staining. **F** Functional behavioral scores, namely, Bederson and grip test, in NK cell-supplemented NRG mice, compared with controls, are shown

To identify a more suitable mouse model for investigating immune cell subset-specific effects in stroke development, we used NRG mice carrying double genetic deficits in the Rag1 and the IL-2-receptor-γ-chain genes. Immunological characterization of NRG mice revealed that mature murine T and B cell populations required for adaptive immunity were absent. NRG mice also displayed diminished numbers of NK and NKT cells (Fig. 4A +  Supplement Fig. 4A + B, WT: n = 6, NRG: n = 7; *p* < *0.001*), which corresponds to previous results [19, 30]. To further substantiate our hypothesis that NK cells should be considered in the interpretation of immune-cell transfer models, NRG mice were reconstituted with NK cells from WT or Rag1^−/−^ mice 24 h prior to tMCAO. For this, cells were isolated by sequential MACS and flow cytometric cell sorting achieving a purity of ≥ 90% (Fig. 4B). NK cell reconstitution from WT reversed the stroke-protective effect observed in NRG mice and resulted in infarctions similar to Rag1^−/−^ controls (Fig. 4C, NRG ctr. 29.4 ± 5.8mm^3^ vs. NRG NK^WT+^ 53.5 ± 9.3mm^3^; *p* < *0.05*; ctr. n = 11; NRG + NK^WT+^: n = 10). Corresponding results were achieved when supplementing NK cells derived from Rag1^−/−^ mice in NRG mice (Fig. 4E, NRG ctr. 56.15 ± 10.02mm^3^ vs. NRG NK^Rag+^ 121.6 ± 7.7mm^3^; *p* < *0.01*, n = 5)*.* In both settings, the increase in infarct size was functionally relevant, since the Bederson score was significantly increased in NRG mice supplemented with NK cells from WT (Fig. 4D, 1.90 ± 0.21 vs. 2.70 ± 0.15; *p* < *0.05*) or Rag1^−/−^ mice (Fig. 4F, 1.80 ± 0.37 vs. 2.60 ± 0.244; *p* = *0.1*).

## Discussion

Rag1^−/−^ mice, lacking mature T and B cells, represent a common adoptive transfer model for immunological stroke research. Several studies used this mouse model to characterize immune cell-specific contributions in stroke development. These studies demonstrated that CD4^+^ and CD8^+^ T lymphocytes [4, 5] contribute to the inflammatory and thrombogenic responses, brain injury, and neurological deficits associated with experimental stroke. In contrast, B cells were shown to have a minor pathophysiological role during acute ischemic stroke [3, 4]. Moreover, Rag1^−/−^ mice were used to characterize the temporal distribution, localization, induction, and function of regulatory T cells in stroke development [31–33], yielding conflicting results. Finally, the Rag1^−/−^ mouse model served as a proof-of-concept experiment to analyze the therapeutic effect of FTY720, a sphingosine-1-phosphate receptor modulator, in models of cerebral ischemia [22] and to determine the contribution of lymphocytes to angiotensin II-induced microvascular dysfunction [34].

However, it is unknown whether NK cell development is altered in Rag1^−/−^ mice and whether persisting NK cell presence and function might preclude immune subset specific analysis, especially of T and B cells. Therefore, the overall goal of this study was to characterize Rag1^−/−^ mice under basal and ischemic conditions with or without NK cells. After the surprising fact that, indeed, Rag1^−/−^ mice still harboring functional NK cells, which had a detrimental effect on the measured stroke outcome, we analyzed and suggest NRG mice as a more appropriate model, to study the influence of specific immune cell population, especially of lymphocyte subsets, on the stroke development.

In ischemic stroke, the paucity of therapeutic options is in strong contrast to the intensive research efforts [35]. Brain-immune-system interaction is considered as a key area to overcome this translational roadblock [21]. Hence, basic research aims to provide a better characterization of the time-dependent contribution of individual immune cells to stroke pathophysiology. Therefore, different mouse models and experimental settings have been implemented in the analysis of time-defined immune cell effects in the past [5, 33, 36]. In this context, pharmacological in vivo immune cell depletion competes with adoptive transfer models in various pathological conditions [7, 10, 37, 38]. Notably, in both settings, depletion of immune cells other than the target cell and persisting and/or compensatory effects by other immune cells must be taken into account when interpreting the results [39].

Despite the marked lymphopenia, our experiments revealed a comparable and functionally equivalent NK cell population in Rag1^−/−^ compared to WT mice. Importantly and in line with previous published results [7], we further observed that depletion of NK cells by an anti-NK1.1 antibody (PK136) in WT and Rag1^−/−^ mice diminished brain infarction in the experimental mouse model. This reduction coincided with a functionally relevant improvement in behavioral scores, suggesting that NK cells might favor cerebral infarction independently of T and B cells.

Since NKT also express NK1.1, PK136 treatment might affect these cells and off-target effects might be involved. NKT cells are lymphocytes that are placed at the intersection between the innate and adaptive immune system, expressing both a T cell receptor, and surface receptors for NK cells [40]. To address this issue, we conducted a proof-of-principle adoptive transfer experiment of NK cells by using the NRG murine strain in experimental ischemic stroke. We reconstituted NRG mice with NK cells from either Rag1^−/−^ or WT mice. Although the separate control cohorts differ slightly in the infarct volume (29.4 ± 5.8mm^3^ vs. 56.15 ± 10.02mm^3^), in both cases NRG animals supplemented with NK cells were significantly more susceptible to acute tMCAO, indicating NK cells as a key lymphocyte determinant of brain infarct size in stroke. As a possible cause of the infarct volume variance in the control groups, it has to be stated that the experiments were not performed at the same day, due to the time-consuming process and the amount of WT or Rag1^−/−^ animals needed as donor throughout the NK cell sorting. Moreover, whether the effect of NK cell substitution is also true at later stages of ischemic stroke (e.g., during the recovery phase) needs to be further addressed.

Although our results further hint towards a critical role of NK cells in ischemic stroke, this study was not designed to provide mechanistic insights, but rather to identify an appropriate adoptive transfer mouse model. This has direct translational implications for human stroke, as stroke-induced neuroinflammation is considered a promising area of research to overcome translational roadblock [35]. However, a multitude of “neuroprotective” agents have shown promising preclinical results without benefiting patients. By using the most appropriate animal model in preclinical studies to identify promising immune targets or to evaluate the efficacy of specific treatments, we likely reduce treatment failures in humans. In this context, we consider the NRG mouse model to be superior to the Rag1^−/−^ model, for in-depth characterization of T and B lymphocytes as well as NK cells in ischemic stroke. Moreover, it has to be noted that the present finding of a detrimental role for NK cells in ischemic brain injury is consistent with previous studies in human stroke. Here, NK cells have been shown to infiltrate ischemic lesions of the human brain, promoting inflammation and neuronal cytotoxicity [7, 10, 37].

Therefore, future studies to thoroughly characterize the underlying mechanism leading to NK cell-mediated neuronal cell death are essential to determine whether NK cells are promising therapeutic targets for ischemic stroke.

## Conclusion

To conclude, we were able to identify a suitable mouse model for the in-depth characterization of individual cell subset-mediated neuroinflammation in stroke. Moreover, we pointed towards a crucial participation of Rag1^−/−^ NK cells in tissue injury resulting from transient focal ischemia and reperfusion of mouse brain. With NRG mice as new adoptive mouse model, we might be able to successfully translate immunological findings from experimental stroke studies to clinical significance that could pave the way for future successful stroke therapies.

## Supplementary Information

Below is the link to the electronic supplementary material.

Figure S1 Scheme on the used mouse strains. Recombination activating gene 1 (Rag1) is a protein that in humans is encoded by the Rag1 gene. The protein encoded by this gene is involved in antibody and T-cell receptor V(D)J recombination, by recognizing recombination signal sequences that flank the V, D and J regions in the genes that encode the heavy and light chains of antibodies and components of T-cell receptors. Because of these effects, Rag1 deletion is used in mouse models (the Rag1-/- and the NOD-Rag1nullIL2rgnull (NRG) mouse model) to impair T cell and B cell development, and functionally deletes mature T and B cells from the immune system. In addition to the Rag1 mutation, NRG mice have a mutation in the interleukin-2 receptor-γ (IL2RG) gene, which encodes the common cytokine receptor γ chain. The γ chain acts as a signal-transducing subunit of cytokine receptors that are essential in the ontogeny and function of lymphocytes, resulting in a lack of l T, B, and NK cells. (PDF 311 KB)

Figure S2 Extensive immunophenotyping of Rag1-/- natural killer (NK) cell subtypes isolated from the spleen. A–C: Using flow cytometry, different NK cell markers were analyzed. A: NK cells, B: iNK cells and C: mNK cells were derived from freshly isolated splenocytes of WT and Rag1-/- mice. Indicated NK cell surface markers are presented as proportions of NK/iNK or mNK cell numbers and as median fluorescent intensity (MFI). (PDF 468 KB)

Figure S3 Immunofluorescence staining of Nkp46-positive NK cells in the ischemic hemisphere. Representative immunofluorescence staining of NKp46-positive NK cells and NeuN-positive neurons in Rag1-/- and WT mice following transient middle cerebral artery occlusion. Nkp46 is stained in red (AF 594), NeuN-positive neurons are depicted in green (AF 488) and the nucleus is shown using DAPI (blue). A: NK cells are consistently found in the ischemic hemisphere of WT and Rag1-/- mice, 24 hours after stroke. NK cells (arrow) often accumulate in close proximity to ischemic neurons (*). High intensity plots and corresponding z-stacks are shown. B: Occasionally, a NeuN signal is found in the Nkp46-positive NK cells (arrow) as depicted by the high intensity plots and the corresponding z-stacks. C: Nkp46-positive NK cells are further located in close spatial proximity to axons. (PDF 272 KB)

Figure S4 Immunophenotyping of NRG compared to wild-type (WT, C57BL/6) mice. A: Proportions of lymphocytes (lymph.), monocytes (mono.), and granulocytes (granulo.) given as the percentage of total CD45high leucocytes were determined by flow cytometry in freshly isolated splenocytes of NRG and WT mice. B: Using flow cytometry, lymphocytes were further subdivided into T and B cells, natural killer (NK) cells, natural killer T (NKT) cells, and γδ-T cells. (PDF 214 KB)

## Data Availability

Data will be shared with qualified investigators upon request, please contact leoni.rolfes@ukmuenster.de.
