# Supplementary figures and images for: Natural Killer Cells Are Present in Rag1−/− Mice and Promote Tissue Damage During the Acute Phase of Ischemic Stroke
Source: Transl Stroke Res. 2021 Jun 8;13(1):197–211. doi: 10.1007/s12975-021-00923-3 (PMC8766401; doi:10.1007/s12975-021-00923-3)

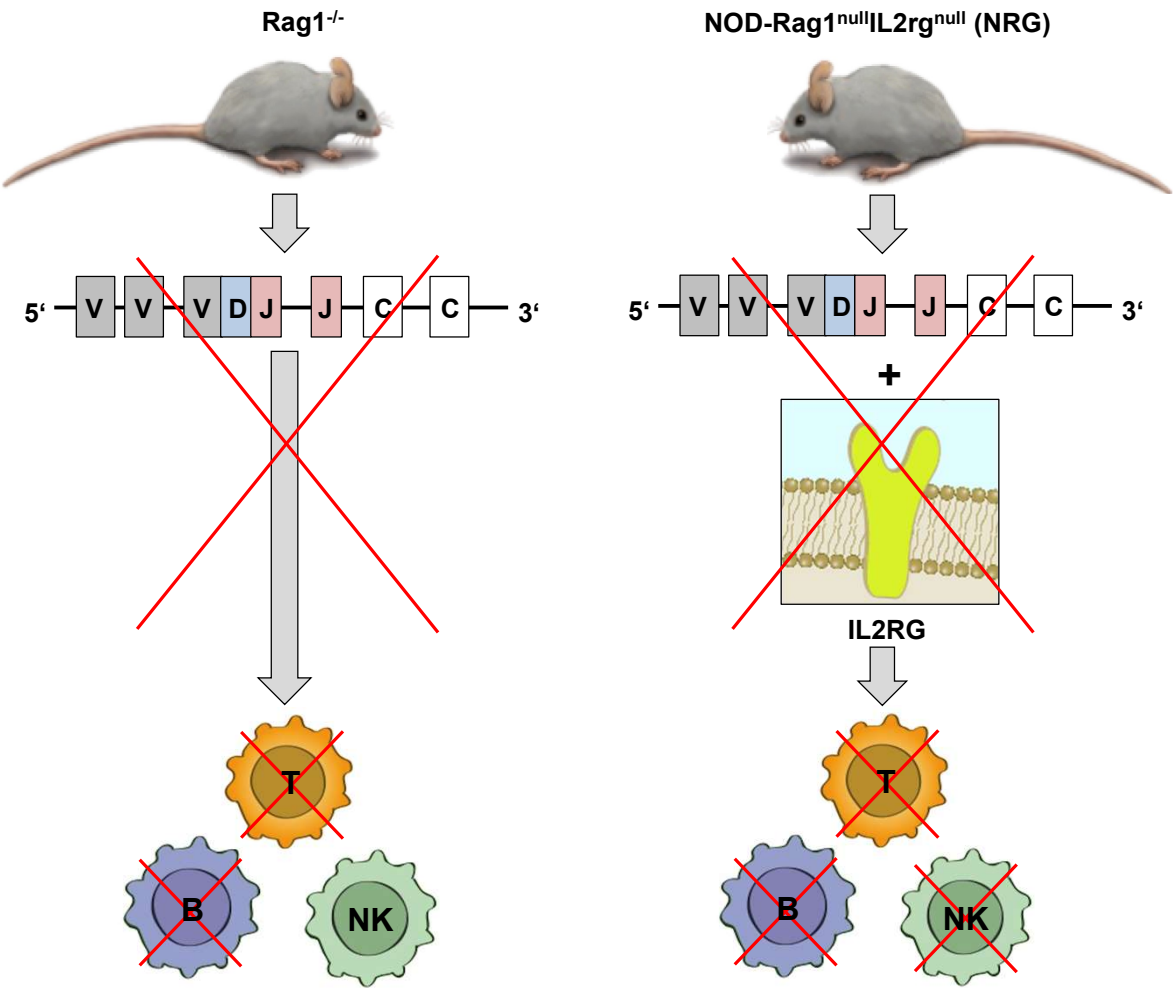

Supplement – Fig. S1

Supplement: Supplementary file 1 — Scheme on the used mouse strains. Recombination activating gene 1 (Rag1) is a protein that in humans is encoded by the Rag1 gene. The protein encoded by this gene is involved in antibody and T-cell receptor V(D)J recombination, by recognizing recombination signal sequences that flank the V, D and J regions in the genes that encode the heavy and light chains of antibodies and components of T-cell receptors. Because of these effects, Rag1 deletion is used in mouse models (the Rag1-/- and the NOD-Rag1nullIL2rgnull (NRG) mouse model) to impair T cell and B cell development, and functionally deletes mature T and B cells from the immune system. In addition to the Rag1 mutation, NRG mice have a mutation in the interleukin-2 receptor-γ (IL2RG) gene, which encodes the common cytokine receptor γ chain. The γ chain acts as a signal-transducing subunit of cytokine receptors that are essential in the ontogeny and function of lymphocytes, resulting in a lack of l T, B, and NK cells. (PDF 311 KB) [file 12975_2021_923_MOESM1_ESM.pdf]

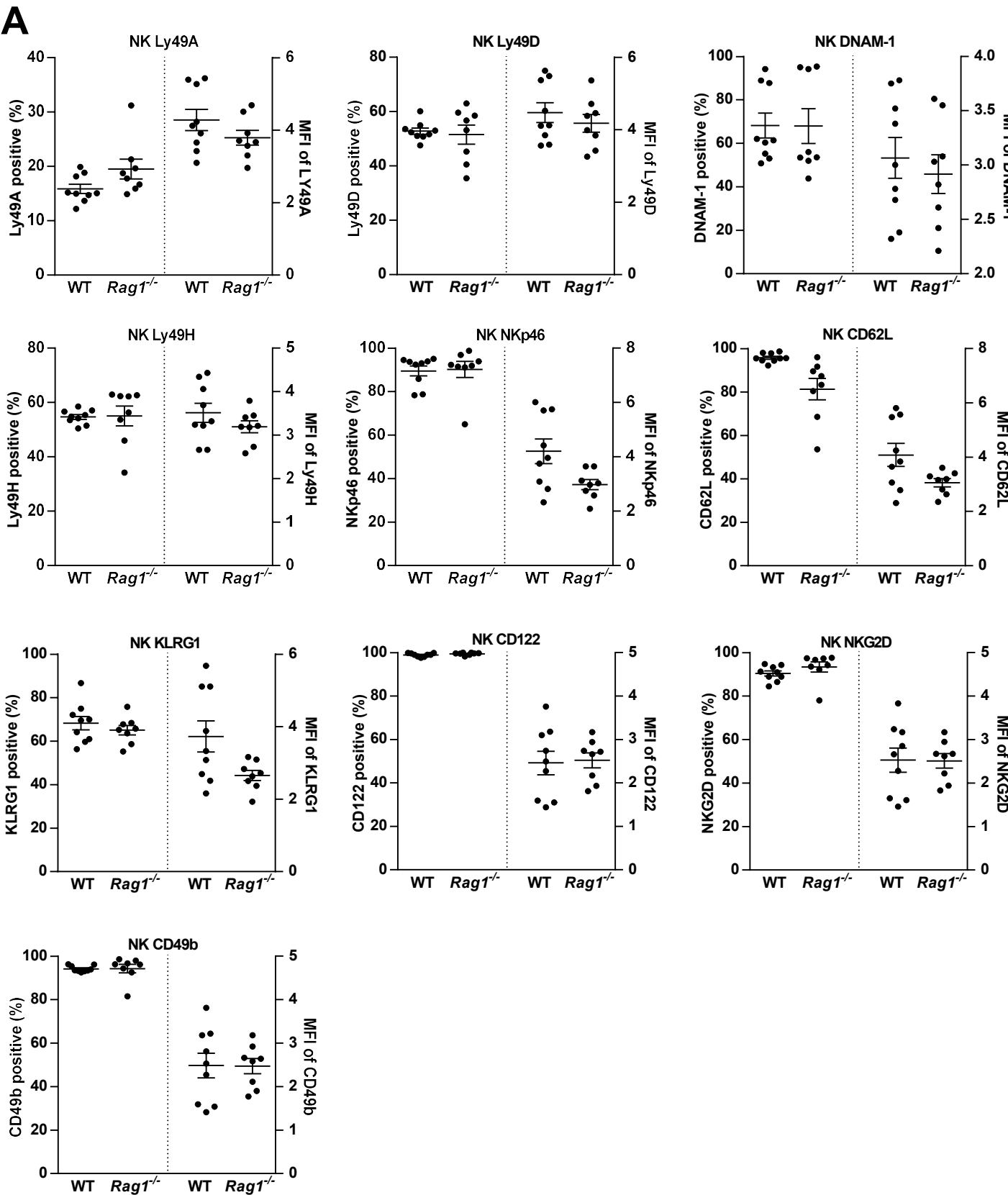

Supplement – Fig. S2

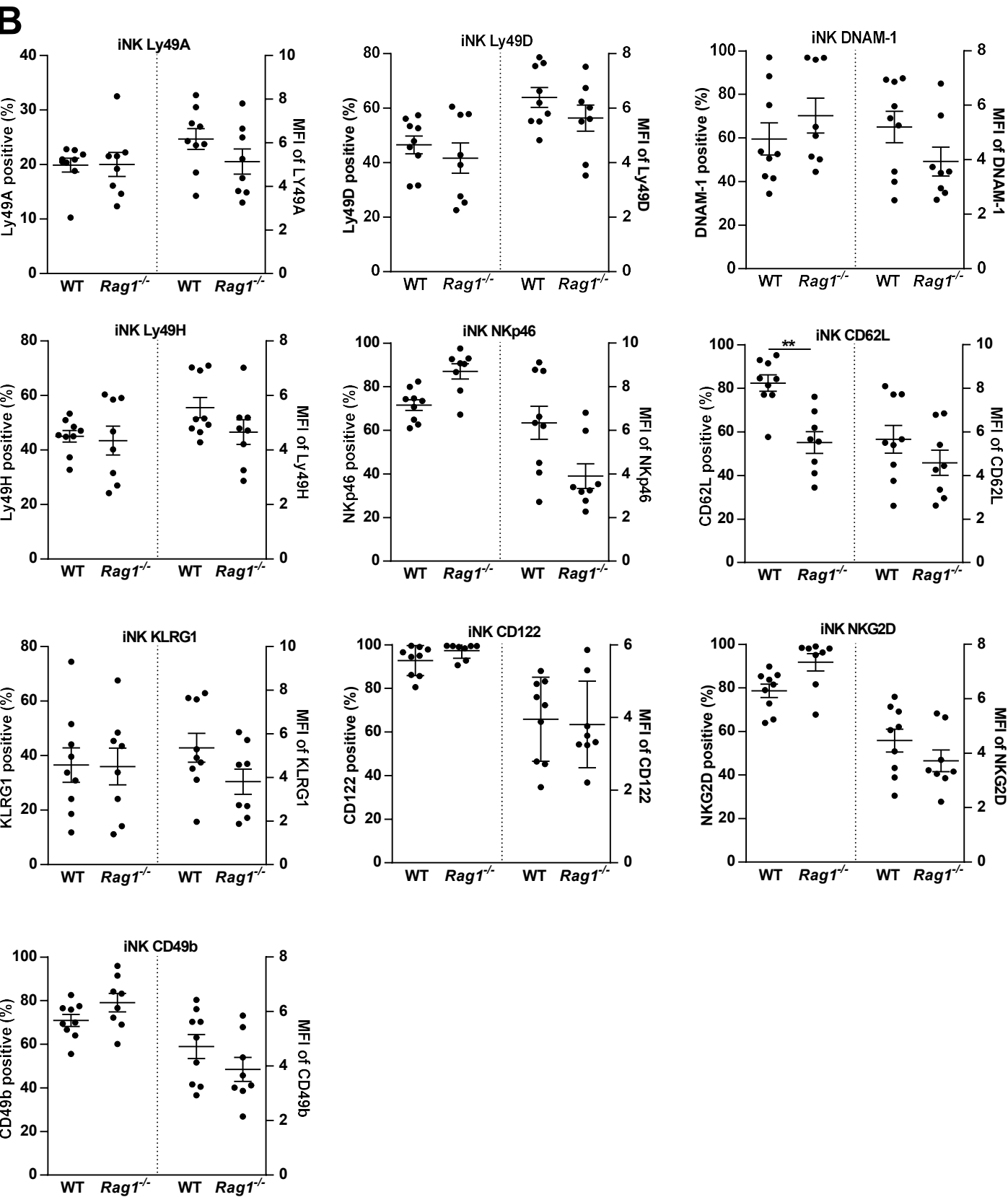

Supplement – Fig. S2

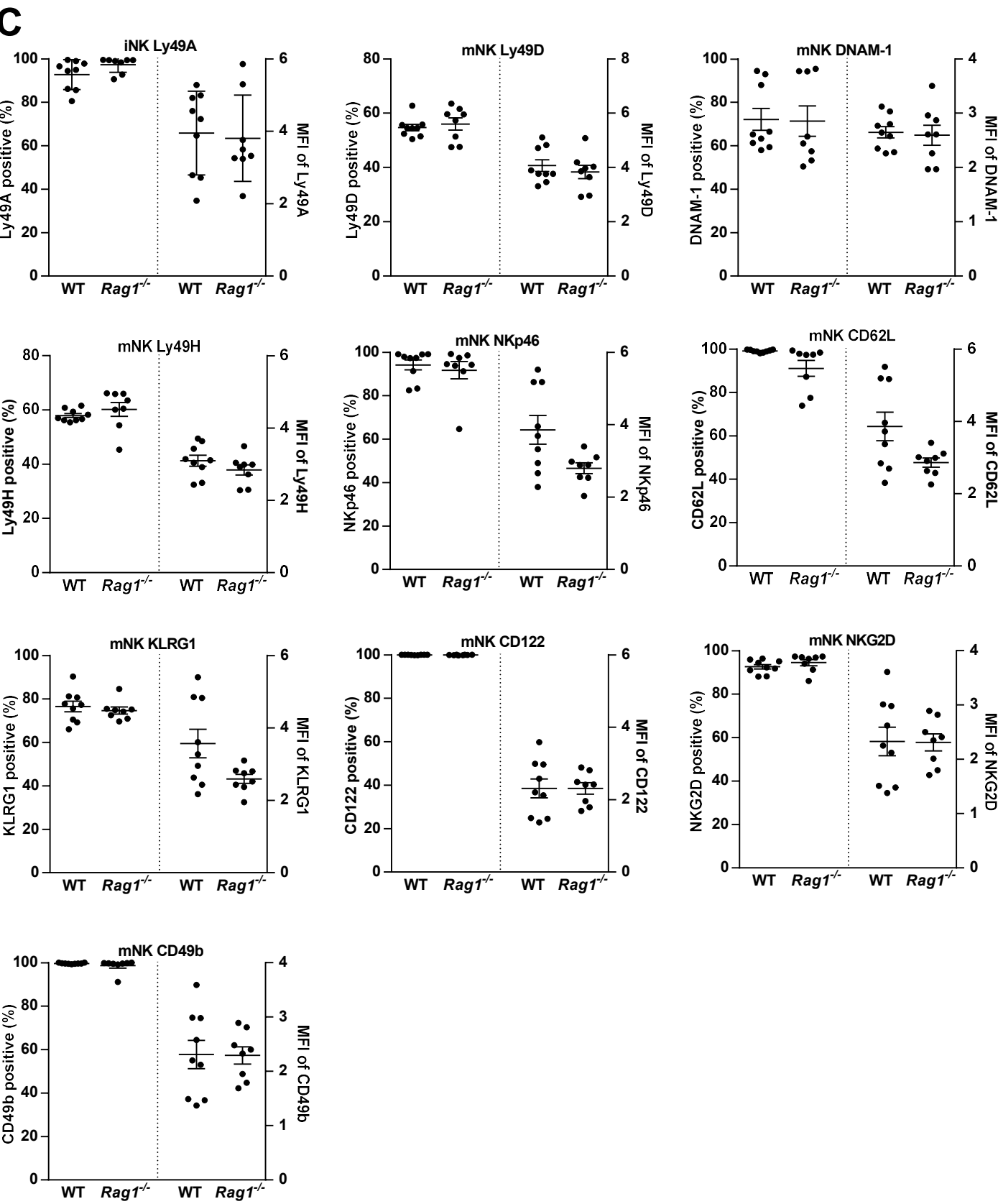

Supplement – Fig. S2

Supplement: Supplementary file 2 — Extensive immunophenotyping of Rag1-/- natural killer (NK) cell subtypes isolated from the spleen. A–C: Using flow cytometry, different NK cell markers were analyzed. A: NK cells, B: iNK cells and C: mNK cells were derived from freshly isolated splenocytes of WT and Rag1-/- mice. Indicated NK cell surface markers are presented as proportions of NK/iNK or mNK cell numbers and as median fluorescent intensity (MFI). (PDF 468 KB) [file 12975_2021_923_MOESM2_ESM.pdf]

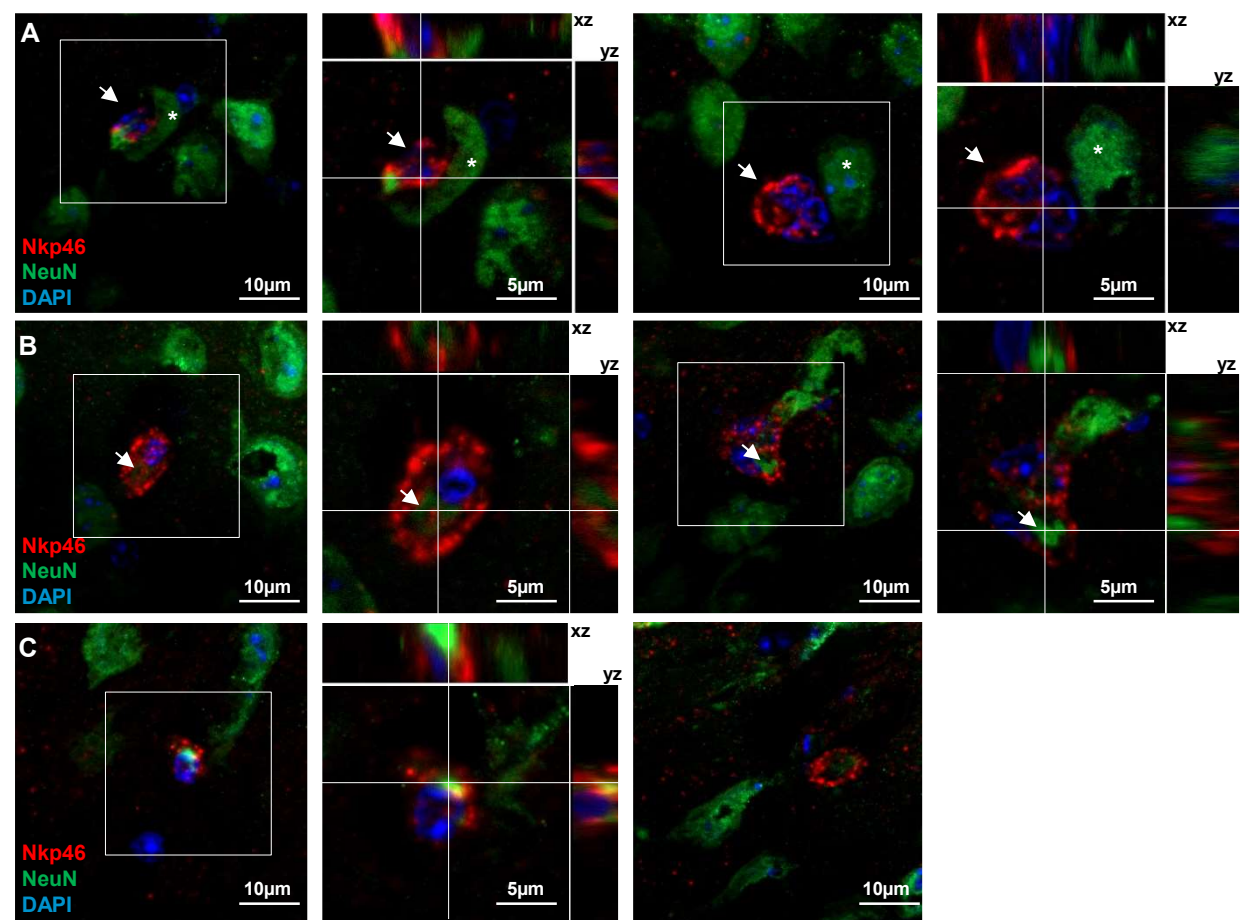

Supplement – Fig. S3

Supplement: Supplementary file 3 — Immunofluorescence staining of Nkp46-positive NK cells in the ischemic hemisphere. Representative immunofluorescence staining of NKp46-positive NK cells and NeuN-positive neurons in Rag1-/- and WT mice following transient middle cerebral artery occlusion. Nkp46 is stained in red (AF 594), NeuN-positive neurons are depicted in green (AF 488) and the nucleus is shown using DAPI (blue). A: NK cells are consistently found in the ischemic hemisphere of WT and Rag1-/- mice, 24 hours after stroke. NK cells (arrow) often accumulate in close proximity to ischemic neurons (*). High intensity plots and corresponding z-stacks are shown. B: Occasionally, a NeuN signal is found in the Nkp46-positive NK cells (arrow) as depicted by the high intensity plots and the corresponding z-stacks. C: Nkp46-positive NK cells are further located in close spatial proximity to axons. (PDF 272 KB) [file 12975_2021_923_MOESM3_ESM.pdf]

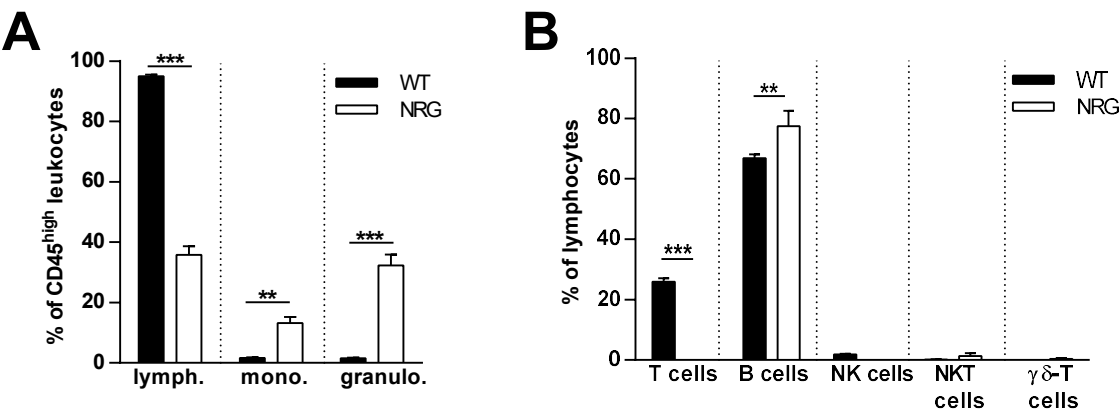

Supplement – Fig. S4

Supplement: Supplementary file 4 — Immunophenotyping of NRG compared to wild-type (WT, C57BL/6) mice. A: Proportions of lymphocytes (lymph.), monocytes (mono.), and granulocytes (granulo.) given as the percentage of total CD45high leucocytes were determined by flow cytometry in freshly isolated splenocytes of NRG and WT mice. B: Using flow cytometry, lymphocytes were further subdivided into T and B cells, natural killer (NK) cells, natural killer T (NKT) cells, and γδ-T cells. (PDF 214 KB) [file 12975_2021_923_MOESM4_ESM.pdf]
